# VoroMQA web server for assessing three-dimensional structures of proteins and protein complexes

**DOI:** 10.1093/nar/gkz367

**Published:** 2019-05-10

**Authors:** Kliment Olechnovič, Česlovas Venclovas

**Affiliations:** Institute of Biotechnology, Life Sciences Center, Vilnius University, Saulėtekio av. 7, Vilnius LT-10257, Lithuania

## Abstract

The VoroMQA (Voronoi tessellation-based Model Quality Assessment) web server is dedicated to the estimation of protein structure quality, a common step in selecting realistic and most accurate computational models and in validating experimental structures. As an input, the VoroMQA web server accepts one or more protein structures in PDB format. Input structures may be either monomeric proteins or multimeric protein complexes. For every input structure, the server provides both global and local (per-residue) scores. Visualization of the local scores along the protein chain is enhanced by providing secondary structure assignment and information on solvent accessibility. A unique feature of the VoroMQA server is the ability to directly assess protein-protein interaction interfaces. If this type of assessment is requested, the web server provides interface quality scores, interface energy estimates, and local scores for residues involved in inter-chain interfaces. VoroMQA, the underlying method of the web server, was extensively tested in recent community-wide CASP and CAPRI experiments. During these experiments VoroMQA showed outstanding performance both in model selection and in estimation of accuracy of local structural regions. The VoroMQA web server is available at http://bioinformatics.ibt.lt/wtsam/voromqa.

## INTRODUCTION

Knowledge of three-dimensional (3D) structures of proteins and protein complexes is essential for comprehensive understanding of protein function, interactions and dynamics. Experimentally determined protein structures are accumulating at a steady pace, however, due to the flood of sequence data, the gap between the known protein sequences and structures is only widening. Not surprisingly, computationally derived structural models of proteins and protein complexes are gaining importance. The usefulness of a computational structural model for specific application is largely determined by the model accuracy (1). Therefore, protein structure assessment methods that are able to provide reliable estimates of both overall model accuracy and accuracy of local structural regions are becoming of prime importance.

One of the practical applications of methods for protein structure assessment is to help users to make an informed selection of computational models. At present, there are multiple automatic modeling pipelines, often implemented as web servers, that can computationally derive structural model(s) for virtually any protein sequence (2). However, having a set of computational models, often significantly different from each other, it may be not at all obvious, which model to select and which regions in the selected model are reliable and which are not. Effective structure assessment methods can help answer such questions.

Assessment of protein structure quality is also a key component in protein structure prediction and refinement. Community-wide CASP experiments that monitor state-of-the-art in protein structure prediction have recognized that the estimation of model accuracy continues to be an important bottleneck (3). Model accuracy estimation is also part of CAMEO, a platform for continuous testing of the computational methods (4).

Protein structures solved using experimental techniques (X-ray crystallography, NMR or cryo-EM) are often considered as the standard of truth. However, it is important to keep in mind that these structures are also models, even though they are derived from experimental data. Although experimental structures deposited in PDB undergo careful validation (5), some of them, especially at low resolution, may occasionally have significant errors such as incorrect chain topology or a shift in the residue register. Structure quality assessment methods can help identify such cases.

Here, we describe the VoroMQA web server devoted to the assessment of protein structure quality. VoroMQA (6), which stands for Voronoi tessellation-based Model Quality Assessment, is a method for the estimation of protein structure quality with an all-atom knowledge-based statistical potential at its core. However, in contrast to traditional statistical potentials based on interatomic distances, VoroMQA characterizes interactions through interatomic contact areas derived from the Voronoi tessellation of atomic balls (7). The VoroMQA scoring function can assess both monomeric proteins and multisubunit complexes. It produces quality scores at the level of atoms, residues and the entire structure. Since VoroMQA is based on contact areas, it can also provide scores for interaction surfaces in a straightforward way. Thus, VoroMQA can directly assess a protein-protein interaction interface, a surface defined by contact areas between atoms from different subunits. Although a number of structure quality assessment methods can score protein complexes, to our knowledge, the ability to provide scores specifically for the protein-protein interface is a unique feature among such methods. The server is designed to provide users not only with an easy access to all the major functionalities of VoroMQA, but also with the ability to interactively control the extent and type of data displayed.

## MATERIALS AND METHODS

### Contact areas

**Figure 1.**
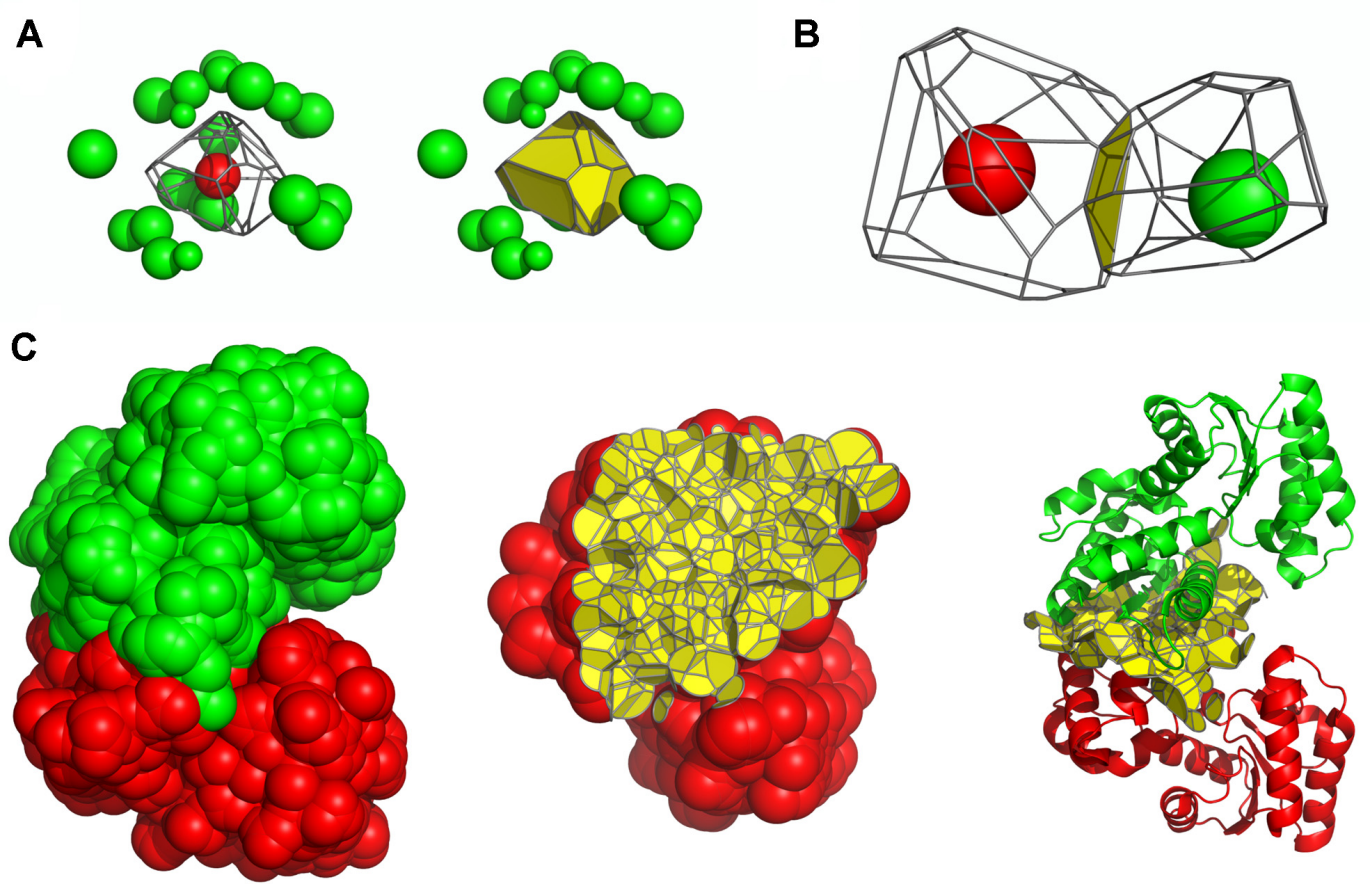
Using Voronoi tessellation to define contacts. (**A**) An example of a Voronoi cell, drawn with edges and with faces. (**B**) Defining a contact as a Voronoi face between two adjacent Voronoi cells. (**C**) Constraining contacts by a solvent accessible surface and describing an inter-chain interface.

A protein structure can be represented as a set of atomic balls, each ball having a van der Waals radius corresponding to the atom type. A ball can be assigned a region of space that contains all the points that are closer (or equally close) to that ball than to any other. Such a region is called a Voronoi cell (Figure 1A) and the partitioning of space into Voronoi cells is called Voronoi tessellation or Voronoi diagram. Two adjacent Voronoi cells share a set of points that form a surface called a Voronoi face (Figure 1B). A Voronoi face can be viewed as a geometric representation of a contact between two atoms. The Voronoi cells of atomic balls may be constrained inside the boundaries defined by the solvent accessible surface of the same balls. Combining constrained contacts can be used to precisely define complex interaction interfaces (Figure 1C). The procedure to construct the described contact surfaces is implemented in Voronota (7), it uses triangulated representations of Voronoi faces and spherical surfaces. Contact areas are calculated as the areas of the corresponding triangulations.

### Scoring using contact areas

VoroMQA (6) evaluates the quality of protein structural models using inter-atomic and solvent contact areas and employing the idea of a knowledge-based statistical potential. A contact type (*a*_*i*_, *a*_*j*_, *c*_*k*_) is described by two atom types (*a*_*i*_, *a*_*j*_) and a contact category *c*_*k*_ and can be assigned a pseudo-energy value *E*(*a*_*i*_, *a*_*j*_, *c*_*k*_) calculated from the corresponding expected and observed probabilities (the probability values are estimated empirically using the contact area values calculated for a set of high-quality experimentally determined protein structures):

(1) \begin{equation*} E(a_i, a_j, c_k) = \log \frac{P_{\rm expected}(a_i, a_j, c_k)}{P_{\rm observed}(a_i, a_j, c_k)} \end{equation*}

Given a single atom ϕ and the set of associated contacts Ω_ϕ_, a normalized pseudo-energy value *E*_*n*_(Ω_ϕ_) is computed using the contact types and areas known for each contact ω ∈ Ω_ϕ_:

(2) \begin{equation*} E_{\rm n}(\Omega _{\phi }) = \frac{\sum _{\omega \in \Omega _{\phi }} E({\rm type}_{\omega }) \cdot {\rm area}_{\omega }}{\sum _{\omega \in \Omega _{\phi }} {\rm area}_{\omega }} \end{equation*}

$E_{\rm n}$(Ω_ϕ_) is transformed into an atom quality score *Q*_a_(Ω_ϕ_) ∈ [0, 1] using the Gauss error function with atom type-dependent μ (mean) and σ (standard deviation) values:

(3) \begin{equation*} Q_{\rm a}(\Omega _{\phi }) = \frac{1}{2} \left( 1 + {\rm erf}\left( \frac{E_{\rm n}(\Omega _{\phi })-\mu _{{\rm type}_{\phi }}}{\sigma _{{\rm type}_{\phi }} \sqrt{2}} \right) \right) \end{equation*}

Given a set of all the atoms in a protein structure, the global structure quality score is defined as a weighted arithmetic mean of the atomic quality scores with weights indicating how deep each atom is buried inside a structure. The quality score of a residue is defined as an average of the quality scores of its atoms.

The score of an inter-chain interface is defined as an average of the quality scores of all the atoms that participate in the inter-chain contacts. Another VoroMQA-based interface assessment measure, called ‘interface energy’ (8), is defined as a total sum of the interface contact areas multiplied by the corresponding pseudo-energy values.

## WEB SERVER DESCRIPTION

### Input

As an input, the VoroMQA web server accepts one or more protein structure files in the PDB format. All non-protein atoms (ligands, nucleic acids, etc.) in input files are ignored. A user can specify either of the two ways to read each of the uploaded PDB files: as plain structures (read all the protein atoms and, if there are multiple ‘MODEL’ blocks, split the input into multiple structures); or as biological assemblies (read all the protein atoms and, if there are multiple ‘MODEL’ blocks, combine the input into a single multimeric structure). Alternatively, a user may request the VoroMQA server to download structures directly from the Protein Data Bank (9) by specifying a PDB ID.

An input structure can be a single subunit or a protein complex comprised of multiple chains. The VoroMQA server performs the same whole structure assessment for both single-chain and multi-chain structures, that is, inter-chain contacts are treated in the same way as contacts within a single chain. However, if requested by a user (via the designated check-box) the server can additionally evaluate all the inter-chain interfaces found in the input structures. In that case the results of interface assessment are appended to the whole structure assessment results.

**Figure 2.**
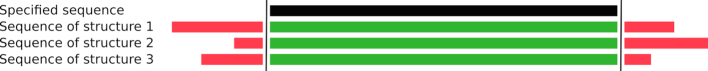
Truncating input structures according to the user-provided sequence. Unaligned regions (red) are removed from the input structures.

In the case of uploading files as plain structures, a user can optionally provide an amino acid sequence to filter and renumber the residues in the input files. If this option is used, then, for each input structure, the VoroMQA server aligns the sequence extracted from the structure with the user-specified sequence. In the case of a multi-chain input structure, sequences of the individual chains are concatenated into a single sequence which is then aligned with the user-specified sequence. The residues that are left unmatched in the resulting alignment are discarded from the input structure (Figure 2). The remaining residues are renumbered according to the user-specified sequence, but the chain names are left unchanged. The user-specified sequence is not used in the scoring process, it is only used to alter (cut and renumber) the input structures. This option removes the need to edit PDB files in cases such as the assessment of a common structural core of models having heterogeneous tails.

### Structure scoring output (default)

**Figure 3.**
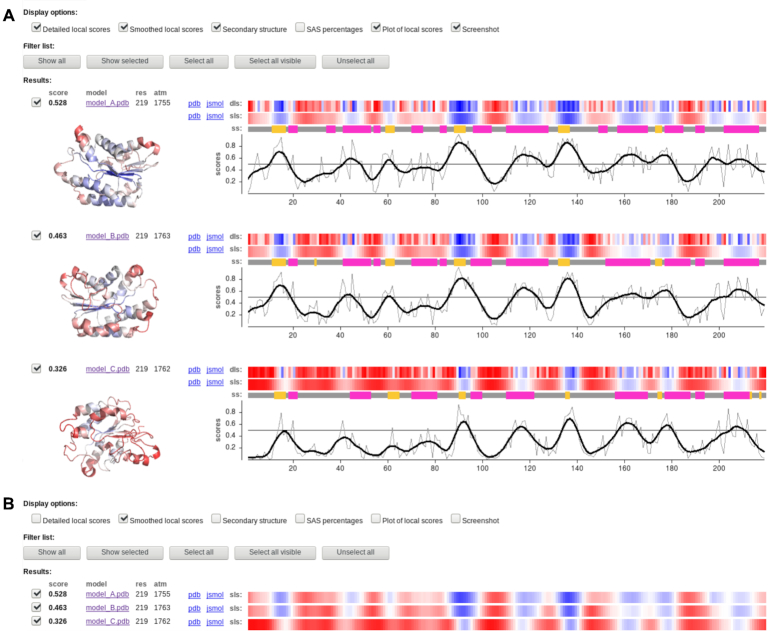
Default output page with global and local scores: default view (**A**); compacted view (**B**).

There are two parts of the output information provided by the VoroMQA server: global and local. The default output page shows both parts alongside (Figure 3).

For every processed structure, the global output contains the global VoroMQA score and the numbers of residues and atoms. To aid users in interpreting the global output, the output scores are put on the plot that summarizes the distribution of VoroMQA scores of high-quality X-ray structures (Figure 5B). This provides a context for judging the level of realism of the processed structures. It is not uncommon to see the global scores below the red line (indicating poor quality) for computational models, but in the case of experimental structures this calls for caution. The structure may have unusual properties or it may have serious flaws (see Discussion for details on this example).

The local output contains local VoroMQA scores (per-atom and per-residue) and additional per-residue information on secondary structure and solvent accessibility. There are two types of local per-residue scores: raw (detailed) and smoothed via sliding window to reduce noise. The local scores are provided in three forms: (i) as temperature factor values written into PDB files that can be either downloaded or viewed in 3D with JSmol (http://www.jmol.org), (ii) as interactive (clickable) color-coded profiles that show per-residue scores and (iii) as an interactive cartesian plot that shows both raw and smoothed local scores together. The secondary structure and solvent accessibility information is also presented as interactive color-coded profiles. Various visualizations can be turned off and on, allowing a user to focus on some of the features without being distracted by the others, which is particularly useful when viewing results for multiple models on a single page (Figure 3B).

### Interface scoring output

**Figure 4.**
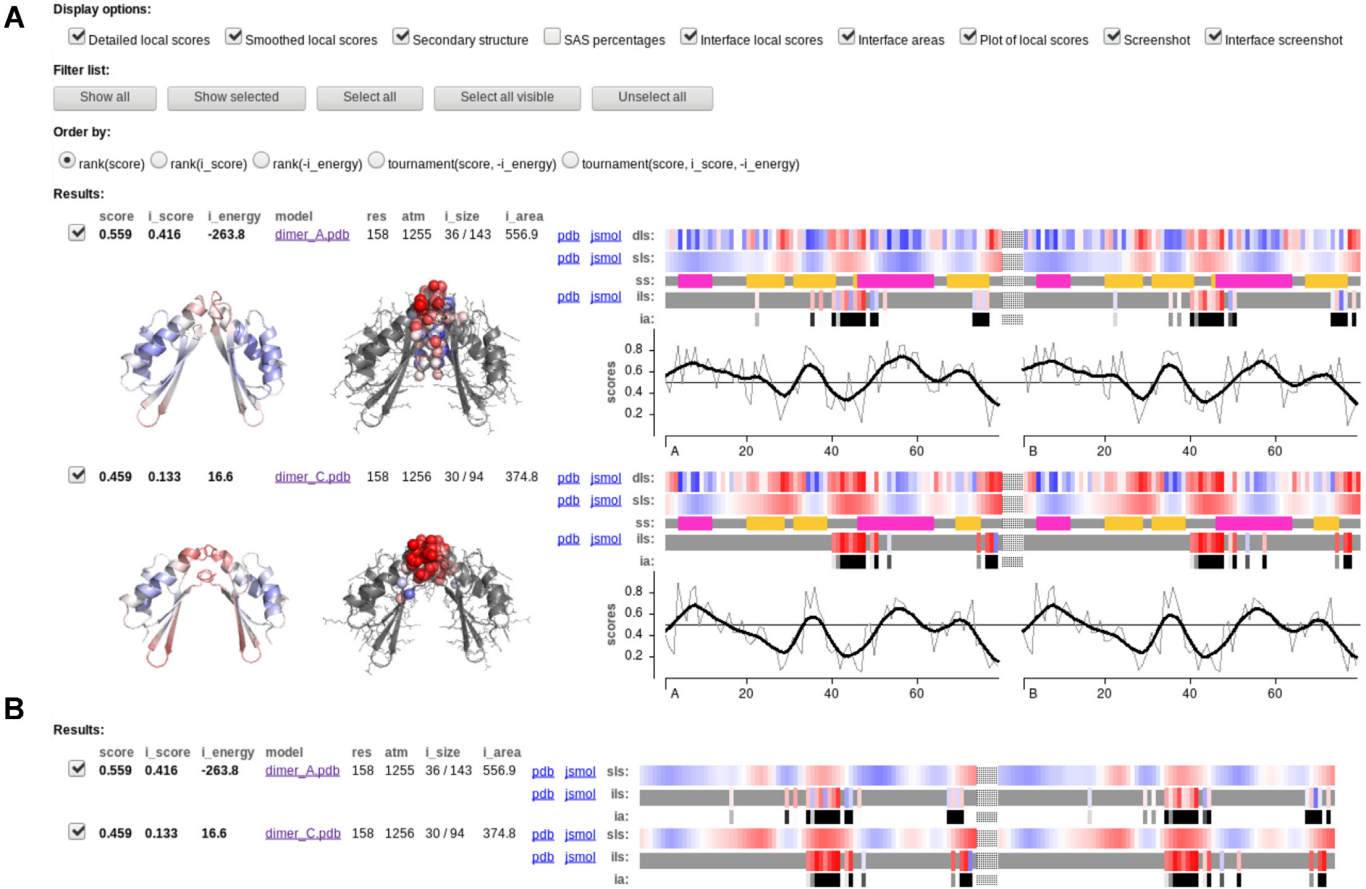
Output page with both whole structure and interface quality assessment results: (**A**) default view; (**B**) compacted view.

If the evaluation of inter-chain interactions was requested, then the output for each processed multisubunit structure is enhanced (Figure 4). The global part is augmented with several values: the numbers of the interface atoms and the interface residues (the atoms and the residues that participate in the inter-chain contacts); the total area of the the inter-chain contacts; the interface quality VoroMQA score (average VoroMQA score of the interface atoms); the total VoroMQA pseudo-energy of the inter-chain contacts.

By default the per-model outputs are sorted by the whole-structure VoroMQA scores. However, there also are other ordering options, including a tournament-based sorting procedure that accounts for both the whole-structure and the interface-based scores (8). Another way for a user to analyze several scores at once is through the interactive chart in which, for every processed complex structure, the whole-structure and the interface-based scores are plotted against the total pseudo-energy of the inter-chain interface.

The local output is augmented with the local VoroMQA scores of the interface atoms and the interface residues. The per-atom scores are written into PDB files and can be viewed in JSmol. The per-residue scores (which are the averages of the per-atom scores) are presented as an interactive color-coded profile. This profile is best viewed in conjunction with the profile that visualizes the inter-chain contact areas of the scored residues (Figure 4B).

## DISCUSSION

The server provides a comfortable interface to the VoroMQA method at the same time enabling a user to perform advanced analysis of scoring results. The VoroMQA server includes the major capabilities exhibited by some of the previously developed protein structure quality assessment servers: producing both global and local quality scores (eQuant (10), ModFOLD (11), ProQ2 (12), ProQ3/ProQ3D (13,14), ProSA-web (15), QMEAN (16,17)); processing multiple structures (eQuant, ModFOLD, ProQ2, ProQ3/ProQ3D); processing structures with multiple chains (DFIRE (18), eQuant, QMEAN, SBROD (19)); providing means to interpret global scores (eQuant, ModFOLD, ProSA-web, QMEAN); providing interactive visualizations of local scores (eQuant, ModFOLD, ProSA-web, QMEAN); taking less than a minute to fully analyze an average-sized input structure (DFIRE, eQuant, ProSA-web, SBROD). In addition to all the aforementioned capabilities, the VoroMQA server allows scoring of inter-chain interfaces. Thus, the server provides quality assessment scores on four levels: atom, residue, interface and whole-structure. This makes the VoroMQA server a uniquely versatile tool for the quality assessment of both monomeric and multimeric protein structures.

An additional factor, contributing to the practical value of the VoroMQA server, is that VoroMQA does not use any additional evolutionary or structural information (e.g. sequence conservation, predicted secondary structure, etc.) that may change with time. Therefore, the same protein structure always produces the same VoroMQA score, a feature important for reproducibility.

Versatility of the server would be of little value without the robust performance of an underlying scoring method. The performance of VoroMQA was tested extensively in recent CASP and CAPRI experiments. In CASP12 the ‘VoroMQA-select’ group, which used VoroMQA to identify the best models generated by automated servers, outperformed all but one group in template-based modeling category (20). The interface scoring by VoroMQA was a key element in achieving the best performance in modeling protein assemblies by the ‘Venclovas’ group during CASP12-CAPRI experiment (8,21). Most recently, during CASP13, VoroMQA was identified as one of the best methods for detection of unreliable local regions, emphasizing its excellent local scoring capabilities (http://predictioncenter.org/casp13/doc/presentations/Assessment_EMA_andRoundTable_Redacted.pdf). The best performance in protein assembly modeling by the ‘Venclovas’ group in CASP13 has reaffirmed the value of interface scoring provided by VoroMQA (http://predictioncenter.org/casp13/doc/presentations/Assessment_assembly_JDuarte.pdf).

**Figure 5.**
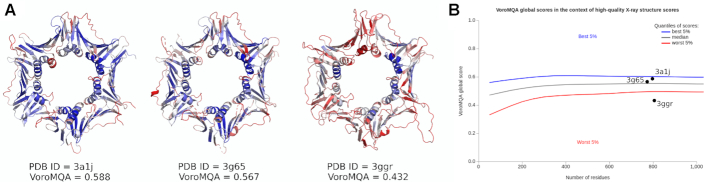
(**A**) Structures of the Rad9-Rad1-Hus1 complex, solved by three different groups, colored by smoothed per-residue VoroMQA scores using red-white-blue color gradient (lower scores are red, higher scores are blue). (**B**) Plot for interpreting global scores for a structure of a given size. The 90% of VoroMQA scores for high quality X-ray structures fall between red and blue lines. The gray line indicates median of the scores. Only 5% of scores fall below the red line and 5% are above the blue line.

Scoring computational models is only one of possible application areas of the VoroMQA server. The server may also be used for independent assessment of experimental structures prior to their deposition into PDB or for helping to avoid utilizing PDB structures that do have serious flaws. The case in point is illustrated with an example in Figure 5. In PDB there are three independently solved structures of the human Rad9–Rad1–Hus1 (9–1–1) DNA damage checkpoint complex (22–24). One of these structures, PDB entry 3GGR (24), is an obvious outlier according to the global VoroMQA scores (Figure 5B). Superposition of 3GGR onto either of the other two structures reveals multiple regions displaying register-shift errors that can be seen as poor (red) local VoroMQA scores (Figure 5A). Unfortunately, this grossly incorrect structure has been used as the basis for other studies, including molecular dynamics simulations (25). VoroMQA and perhaps other methods of similar nature could have easily prevented selection of this incorrect structure as the basis for subsequent studies.

## CONCLUSIONS

The VoroMQA server provides a straightforward way to assess any protein structure of interest, be it experimental or theoretical, monomeric or multimeric. User-friendly interface provides both global and local scores and enables visualization of these scores both in the context of 3D structures and along the sequence. The server therefore might be useful for various tasks such as flagging suspicious experimental structures and pinpointing problematic regions, selecting the most accurate computational models and estimating the accuracy of local regions as well as assessing the interaction interfaces in protein complexes.
